# Changes in homegardens in relocation villages, a case study in the Baiku Yao area in Southern China

**DOI:** 10.1186/s13002-023-00578-4

**Published:** 2023-02-27

**Authors:** Renchuan Hu, Chuangui Xu, You Nong, Binsheng Luo

**Affiliations:** 1 Guangxi Institute of Chinese Medicine & Pharmaceutical Science, Nanning, 530022 China; 2 Guangxi Key Laboratory of Traditional Chinese Medicine Quality Standards, Nanning, 530022 China; 3 Lushan Botanical Garden, Jiangxi Province and Chinese Academy of Sciences, Lushan, 332900 China

**Keywords:** Homegarden, Baiku Yao, Ethnobotany, Traditional knowledge

## Abstract

**Background:**

Baiku Yao is an ancient branch of the Yao people in China who have the custom of maintaining homegardens. The local government has relocated some villagers to improve their livelihood. To study the characteristics of Baiku Yao homegardens and the impact of relocation, we conducted an ethnobotanical study on homegardens in the relocated villages of Huaili and Yaozhai and in the ancient villages of Yaoshan and Duonu from January 2019 to May 2022.

**Method:**

Data on homegarden plants were collected through semi-structured interviews with homegarden households. A total of 4 villages (i.e., two relocated and two ancient villages) were selected for detailed investigation. In each village, 60 homegardens were chosen randomly. In addition, the RFC index and Jaccard value were used to analyze and evaluate the homegarden plants we recorded.

**Result:**

The study recorded a total of 213 species of homegarden plants with approximately 11 functions. Baiku Yao homegardens are small in size but rich in species and functions, and their utilization efficiency is extremely high. The Jaccard value shows that the homegarden plants in Huaili and Yaozhai have the highest similarity. Neighborhood sharing and market purchasing are the two most important sources of local homegarden plants. Additionally, medicine and food are the two most important uses of homegarden plants. Ornamental plants also play a significant part, especially in relocated villages. The comparisons indicate that the diversity of homegarden plants in the investigated ancient villages is better preserved than in relocated villages. Due to frequent exchanges between the villages and the outside world, Yaoshan Village, as an older relocated village, maintains a good traditional culture in its homegardens. As a newly relocated village, Duonu Community has developed a complicated homegarden system with only much less plant diversity. The development of local tourism has also impacted the composition of homegarden plants. The study found that plants such as *Zea mays*, *Morus alba*, and *Capsicum annuum* are closely related to local life and livelihood.

**Conclusion:**

The traditional knowledge of homegarden plants in investigated ancient villiages maintained good diversity and has been affected much less by the modernization and tourism industry development compared to the relocated villages. The composition of homegarden plants is closely connected to the local livelihood. In the future development of Baiku Yao communities, protecting homegarden plant diversity and functional diversity is crucial.

## Introduction

Baiku Yao is a branch of the Yao ethnic group mainly living in Nandan County in Guangxi Province and Libo County in Guizhou Province in China [1]. “Baiku Yao” means “Yao people wearing white pants”; they received this name because the grown men of Baiku Yao wear white pants daily. Many of the Baiku Yao people lived in a mountainous area inaccessible to the modernized world. However, the government recently constructed roads to easily access these isolated mountain villages. This infrastructure construction ultimately opened a corridor for Baiku Yao to exchange goods and information with the outside world. The traffic improvements eventually made considerable changes in Baiku Yao culture, from a completely traditional culture to a direct shift in modern life. We were interested in examining how this transformation from traditional to modern culture changed their lifestyle over time. Our past research on Baiku Yao focused on plants used for various purposes, such as herbal medicine, foraging, dyeing, and veterinary medicine [1–3], and showed that Baiku Yao holds wealthy traditional knowledge about plants and their applications.

The Baiku Yao community lived in mountainous areas for a long time and maintained the traditional livelihood. According to interviews with the local government, with support from the Chinese government's poverty alleviation policy, some Baiku Yao villages in Yaoshan Township in Libo County of Guizhou Province in China were relocated between the 1950s and 2000. In 2009, the local government began to develop tourism on a large scale to create employment and economic development. With the government's support, another Baiku Yao area, Nandan County in Guangxi Province, China, started construction of the “thousand households Yao village” project in 2017, completed it in 2020, and formed a new village dominated by Baiku Yao (accounting for more than 90% of the population), called the Duonu Community. Yaoshan village and Duonu Community, as relocated villages, their residents were moved from different villages. (According to local poverty alleviation policies, low-income families from different villages were encouraged to move out from their original village to reform a new village, Yaoshan village and Duonu Community, and those families were provided new houses and other infrastructure.) The relocation process of Baiku Yao village may have affected their traditional knowledge base. Therefore, comparing relocated and ancient villages could provide a good case for studying the dynamic changes in traditional knowledge during migration.

Several case studies have been conducted among migrants of ethnic groups to understand how relocation and migration affect traditional knowledge and ethnic cultures. Some studies have reported that many migrant groups have started to practice new plant application knowledge to adapt to environmental changes and the availability of the same plant resources at the relocation site [4]. Ecological migration is a phenomenon of population migration due to the interaction of the ecological environment and other factors [5]. Ma et al. (2019) explored changes in the knowledge of traditional forage plants in ecological migrant groups and found considerable differences in the diversity of related knowledge retained by ecological migrants in different regions [5]. The people who migrated to nearby areas from their original settlement retained more knowledge than those who moved far from their native place because of significant differences in natural resources [5]. Moreover,  in the same case, the younger generation mostly forgot about the traditional knowledge of forage grass [5]. Studying the dynamic change in traditional knowledge among migrant groups might help protect their biocultural diversity [6].

Homegarden is usually described as a small agroforestry system in or near local houses that can provide entertainment, food, medicine, income, and other essential functions [7]. Many studies on traditional homegardens are often related to agriculture, ecology, nutrition, botany, and many other fields [8]. Plant diversity, an important core component of homegarden ecology and function, is also the focus of many researchers [9]. Homegarden plants are plants with certain functions purposely preserved or cultivated in homegardens [10]. Usually, homegarden plants have multiple functions, are closely connected to the local livelihood, and can reflect the local culture [11]. Maintaining a homegarden is common practice in many rural communities, including Baiku Yao.

In the case of Baiku Yao, a pilot investigation showed that their homegardens are rich in plant resources and are important carriers of traditional genetic resources and knowledge, guaranteeing the community's livelihood. Thus, we also assumed that homegarden plants could indicate the difference between homegardens in ancient villages and relocated villages in the Baiku Yao area. This might also help explain the dynamic change in related culture and knowledge during the relocation.

In this study, we aimed to (1) determine the plant compositions and special characteristics of Baiku Yao homegarden plants and (2) explore the changes in Baiku Yao's traditional knowledge by comparing the plant compositions in homegardens in relocated and ancient villages. This ethnobotanical study not only helps to document and protect the traditional knowledge of homegarden plant but also provides a reference for protecting the biocultural diversity in other cases of community migration and community relocation in the future.

## Method

### Study area

**Fig. 1:**
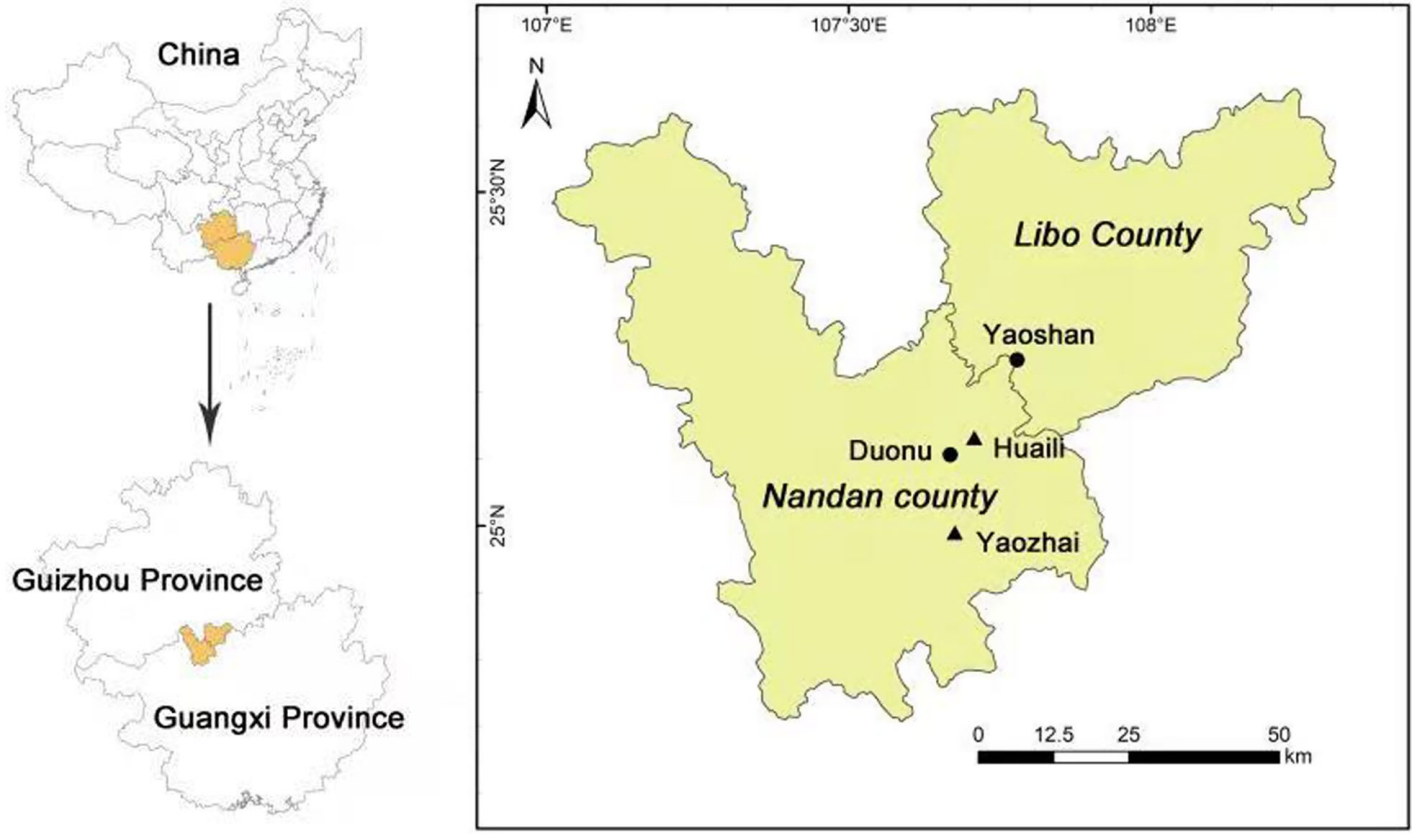
The study area

The Baiku Yao residential area is located on the slope of the transition from the Yunnan-Guizhou Plateau to the Nanling Hills [12]. Its geographical environment features characteristics of both the Yunnan–Guizhou Plateau and the Nanling Mountains [12]. The area has a humid subtropical climate and rich surrounding vegetation species [12]. To explore the impact of relocation on traditional knowledge, we selected two Baiku Yao relocated villages (**Yaoshan Village** in Libo County and **Duonu Community** in Nandan County) and two Baiku Yao traditional ancient villages (**Huaili Village** and **Yaozhai Village** in Nandan County) for detailed investigation (Fig. 1). Huaili Village and Yaozhai Village are the two most traditional and ancient Baiku Yao villages. Both of them have thousands of years of history. The data sources mentioned in the following village introductions are from the local government.

**Yaoshan village** is located in Yaoshan Township, Libo County (107.766609 N; 25.234635E; 514 m asl). This village was formed to relocate the Baiku Yao residents in 1953 to relieve poverty under the national plan. Currently, there are 738 households and 3177 people living in this village. However, due to the higher number of migrants and lower availability of land, most people engage in tourism activities (e.g., performances and exhibitions) to sustain their livelihood. Some migrants have returned to their native places to cultivate food crops and maintain their homegardens.

**Duonu Community** (village) is located in Lihu Township, Nandan County, Guangxi Province (107.653244° N; 25.094444° E; 575 m asl). The relocation of Baiku Yao people to this village started in 2017 and was completed in 2020. A total of 1123 households comprising 5903 people were reallocated to the Duonu Community, most of which were from the poor villages of Baiku Yao, such as Huaili, Dongjia, Yaoli, Badi, and Renguang in Lihu Township. The Duonu Community is located on the slopes of the Masson pine forest. Most houses are constructed close to each other due to limited land availability and high population density, and most villagers depend on the local market to fulfill their daily requirements. However, some old inhabitants return to their native areas to grow food and vegetables to sustain their livelihood.

**Huaili Village** is located on a rocky mountain near Lihu Township (107.693099 N; 25.119346E; 753 m asl), Nandan County, away from the township government. Baiku Yao people dominate the population of this village. A total of 609 households with 3169 people reside in this village. Huaili is one of the ancient villages of Baiku Yao, where these people have lived for more than a thousand years and still maintain their traditional culture. The villagers have sufficient land and produce corn as their primary food.

Like Huaili Village, **Yaozhai** is also a traditional Baiku Yao village (107.658019 N; 24.978054E; 741 m asl) located in Baxu Township, Nandan County. There are 321 households with 1492 residents residing in this village, and the Baiku Yao people dominate 90% of the population of this village. Most of the residents produce rice and corn to sustain their livelihood. However, some residents work as migrant workers.

### Data collection

We conducted a preliminary investigation on the homegarden plants and their role in fulfilling the daily needs of the Baiku Yao communities. A total of 4 villages (i.e., two relocated and two ancient villages) were selected for detailed investigation from January 2019 to May 2022. Given the distribution characteristics of Baiku Yao families in four villages and the validity of data comparison, we numbered the homegardens of each village so that different homegardens corresponded to different numbers. Sixty households (homegardens) were randomly selected in each village by lottery, and appropriate fine-tuning was conducted with the help of local guides. We collected the data using semi-structured face-to-face interviews with homegarden households [13]. Since some Baiku Yao in the study area could not speak fluent Mandarin, assistance was obtained from local guides during the fieldwork. All interviews were conducted in the Baiku Yao language and translated into Mandarin with the help of a local guide. We asked five main questions: (i) what are the main plants in your garden, (ii) what are the functions of these plants, (iii) how do you use this plant (processing methods), (iv) which part is used and (v) where this plant/seed was collected [14, 15]?

We also collected herbarium voucher specimens or took photos during the survey to confirm species identification using Flora of China [16], Flora of Guangxi [17], and botanical Web sites (e.g., http://www.iplant.cn/, http://www.cvh.ac.cn/search, http://ppbc.iplant.cn/). Finally, the identified specimens were reaffirmed by taxonomic experts from the Guangxi Academy of Traditional Chinese Medicine and the inventory of homegarden plants. All specimens were preserved in the Herbarium of the Guangxi Zhuang Autonomous Region, Institute of Traditional Chinese Medicine (GXMI).

### Quantitative analysis

We used the Jaccard index and the RFC index to evaluate the data. The Jaccard index was used to detect the similarity of homegarden plants among the different villages, whereas the RFC index was used to evaluate the importance of homegarden plants in the daily livelihood of Baiku Yao. The Jaccard index is calculated according to the following formula [5, 18]:

$${\mathrm{JI}} = \frac{{\mathrm{C}}}{{{\mathrm{A}} + {\mathrm{B}} - {\mathrm{C}}}} \times 100$$

where A and B represent the number of homegarden plants owned by each of the two villages. C represents the number of homegarden plants in both villages, and the JI value ranges between 0 and 100 [5, 18]. A higher JI value indicates a higher homegarden plant similarity between the two villages [5, 18].

The RFC values were calculated according to the following formula [19]:

$${\mathrm{RFC}} = \frac{{{\mathrm{FCs}}}}{N},$$

where FCs represent the frequency of citation (total number of frequencies mentioned by all respondents to a specific plant), and *N* represents the number of all respondents [19]. In this study, we made some changes. FCs represent the total frequency of a specific plant appearing in the homegardens of the investigated villages, while N represents the total number of homegardens (households) interviewed. The RFC value is between 0 and 1; the higher the RFC, the closer the connection between the homegarden plants and the daily life of Baiku Yao [19].

## Results

### The characteristics of traditional Baiku Yao homegardens

**Fig. 2:**
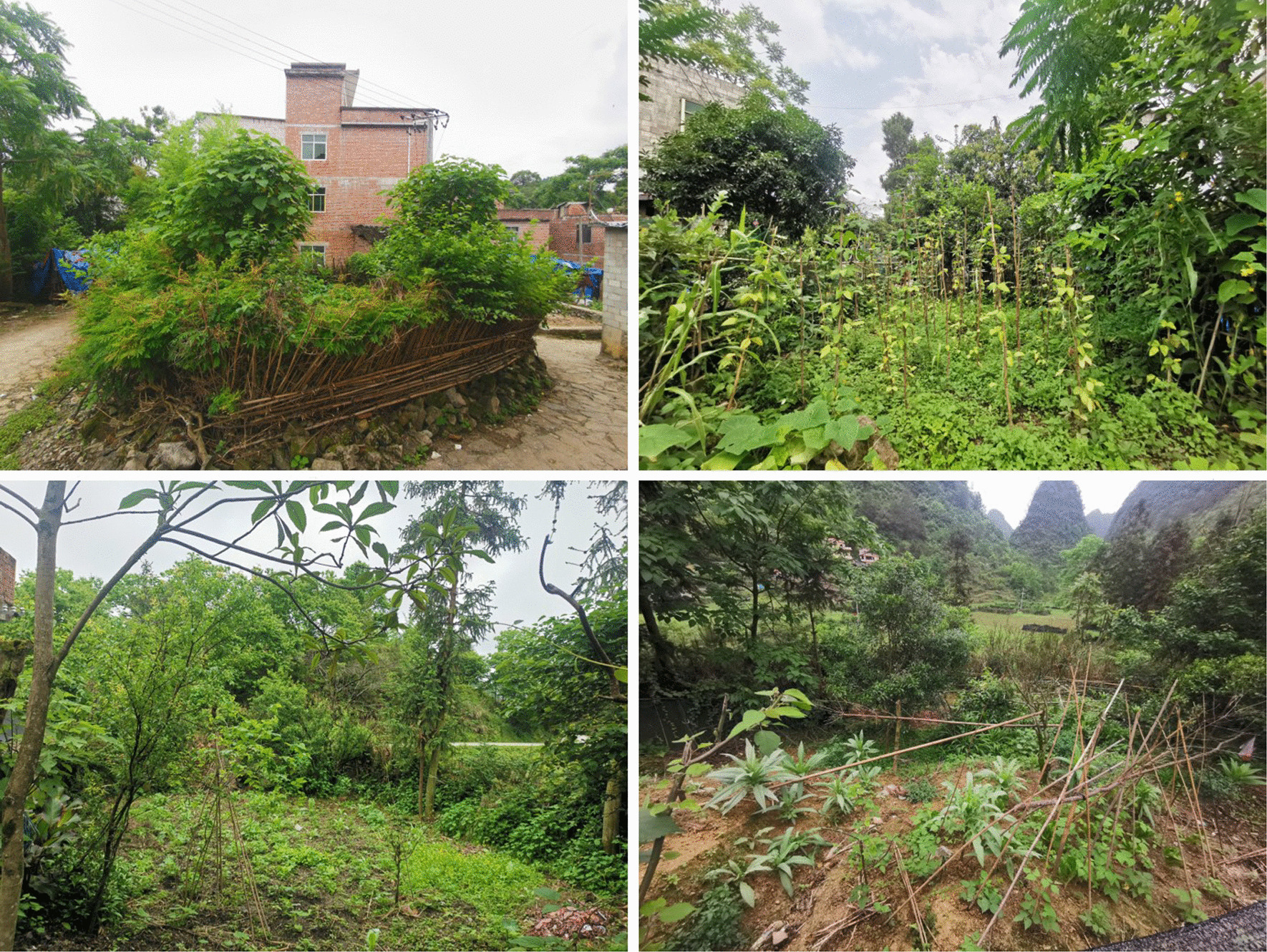
Baiku Yao homegardens

Baiku Yao depends on natural resources for food and other domestic products for their daily needs. Therefore, they greatly respect nature and offer prayers for nature in their daily routine. They frequently use plants like *Caesalpinia decapetala*, *Pterolobium punctatum*, and *Maclura cochinchinensis* to make living fences (Fig. 2) for the homegardens, which they believe the living shrubs are robust. Also, they greatly respect the prominent and old trees growing near the houses (sometimes near or in the homegardens as part of the homegarden systems). Thus, they keep and protect most of the large and old trees at the village entrance or near houses to maintain shade, cool, and landscaping. According to all of our participants, every Baiku Yao believes that big trees next to homes are sacred, and they protect the village and its prosperity. They also think that destroying a sacred tree will destroy the village's Feng shui and negatively affect future generations' well-being. For example, *Keteleeria davidiana* var. *calcarea* tree, usually found near the homegardens or houses, is believed to be inhabited by gods (because its branches are relatively flat and suitable for gods to live). As a national second-class protected plant in China, *Keteleeria davidiana* var. *calcarea* has been particularly well protected in the Baiku Yao area.

Baiku Yao mostly lives in mountainous areas and usually has few land holdings. Thus, their traditional homegardens are also very small (less than 10 square meters on average, Fig. 2). Locally, however, the homegarden space utilization efficiency is extremely high, and the space is clearly structured and hierarchical according to our observations. On the periphery, the fences of homegardens are mainly wild or cultivated plants with certain functions. Local people believe that a living fence is more stable and can increase the space use efficiency of homegardens. For example, planting *Morus alba*, *Broussonetia papyrifera*, and some fruit trees as fences is very common in the Baiku Yao area. In addition, the lower-layer space is often planted with shade-tolerant spices or medicinal herbs. Seasonal vegetables are usually cultivated in the middle area of homegardens. For example, during the winter and the spring, they usually grow *Brassica rapa* subsp. *Campestris*, *Brassica juncea*, and *Spinacia oleracea*, while during the summer, they usually grow *Cucurbita moschata*, *Vigna unguiculata*, *Glycine max*, and *Phaseolus vulgaris*.

The Baiku Yao people are well aware of the importance of maintaining high species diversity in their homegardens to fulfill their daily requirements. According to their living strategy, due to the poor soil and a changeable climate, the benefits of planting a single species may be low or risky, so planting a variety of plants can reduce the risk as much as possible and obtain better returns. The locals also believe that high plant diversity can effectively reduce the harm from pests or plant diseases. The traditional local philosophy of maintaining the homegarden system reflects the wisdom of the Baiku Yao people.

### Plant diversity in Baiku Yao homegardens

As shown in Table 1, 213 species were identified in the homegardens of Baiku Yao, belonging to 172 genera and 80 families. The commonly represented families were Poaceae (15 species), Fabaceae (12 species), Asteraceae (9 species), Rosaceae (8 species), Rutaceae (8 species), and Solanaceae (8 species), most of which are edible and ornamental plants.

**Table 1:** Inventory of homegarden plants in Baiku Yao area

Scientific name	RFC	Voucher ID	Location	Vernacular name	Source	Family name	Life form	Usage	Use part	Use method
*Zea mays* L	0.93	HRC1035	HL, YS, DN, YZ	rong	Market, self-preservation	Poaceae	Herb	Staple food, forage, brewing	Seed, tender leaf	Ground seeds for polenta, cooked seeds for brewing; raw or cooked seeds and tender leaves as forage
*Capsicum annuum* L	0.87	HRC1046	HL, YS, DN, YZ	e biu	Market, self-preservation	Solanaceae	Shrub	Vegetable	Fruit	Stir-fried with meat or making dipping sauce
*Morus alba* L	0.87	HRC1004	HL, YS, DN, YZ	nong wo ze	Neighborhood	Moraceae	Tree	Forage, vegetable, fence, medicine	Root, leaf	Root: decoction to treat parasite and cough; leaf: externally for bone fracture; tender leaf as forage or vegetable
*Eriobotrya japonica* (Thunb.) Lindl	0.68	HRC1005	HL, YS, DN, YZ	pi le	Neighborhood, market	Rosaceae	Tree	Fruit, medicine	Fruit, leaf	Leaf: externally for bone fracture, decoction to treat cough
*Ipomoea batatas* (L.) Lamarck	0.68	HRC105	HL, YZ	yan du	Self-preservation, market	Convolvulaceae	Herb	Forage, vegetable, medicine	Tender leaf	Making soup; raw or cooked as forage; externally used for centipede-bite
*Solanum melongena* L	0.68	HRC1045	HL, YS, YZ	ya gu	Market	Solanaceae	Herb	Vegetable	Fruit	Boiled with other vegetables; roasted to make dipping sauce
*Glycine max* (L.) Merr	0.67	HRC1055	HL, YS, DN, YZ	dao	Market, self-preservation, neighborhood	Fabaceae	Herb	Vegetable, forage	Seed	Stir-fried, boiled, or making tofu
*Phaseolus vulgaris* L	0.66	HRC1024	HL, YS, DN, YZ	dao mu	Self-preservation	Fabaceae	Liana	Vegetable	Fruit	Stir-fried
*Agastache rugosa* (Fisch. et Mey.) O. Ktze	0.65	HRC1015	HL, YS, DN, YZ	ya pang ga ga	Market, neighborhood	Lamiaceae	Herb	Spice	Leaf	Add it when stewing meat
*Cannabis sativa* L	0.65	HRC86	HL, YS, YZ		Neighborhood, self-preservation	Cannabinaceae	Herb	Vegetable, medicine	Seed, tender leaf, whole plant	Seed: grinded for soup; tender leaf: for hotpot; whole plant: medicinal bath for unblocking meridians and treating paralysis
*Brassica rapa* var. *oleifera* de Candolle	0.62	HRC940	HL, YS, DN, YZ		Market	Brassicaceae	Herb	Vegetable, forage	Tender leaf	Eaten after boiled; cut, raw or boiled as forage
*Cucurbita moschata* (Duch. ex Lam.) Duch. ex Poiret	0.62	HRC951	HL, DN, YZ	gao	Self-preservation	Cucurbitaceae	Herb	Forage, vegetable	Fruit, seed, tender leaf	Fruit: cut, raw or boiled as forage; seed: stir-fried as a snack; tender leaf: eaten after boiled
*Zingiber officinale* Roscoe	0.6	HRC1051	HL, YS, YZ	a qiang	Self-preservation, neighborhood	Zingiberaceae	Herb	Spice	Stem tuber	Cook with the meat to remove the meaty smell
*Allium sativum* L	0.58	HRC1052	HL, YS, YZ	wo mie hou	Self-preservation, neighborhood	Amaryllidaceae	Herb	Vegetable	Whole plant	Stir-fried with meat
*Amaranthus tricolor* L	0.58	HRC829	HL, YS, DN, YZ	wo niu	Self-preservation	Amaranthaceae	Herb	Forage, vegetable	Tender branch	Cut, raw or boiled as forage; eaten after boiled
*Fagopyrum dibotrys* (D. Don) Hara	0.57	HRC935	HL, DN, YS, YZ	ya weng	Neighborhood	Polygonaceae	Herb	Vegetable, forage	Tender leaf	Making soup, boiled as forage
*Osmanthus fragrans* (Thunb.) Loureiro	0.57	HRC979	HL, YS, DN, YZ		Primary species	Oleaceae	Tree	Ornamental, medicine	Fruit, flower	Fruit: brewed in wine to nourish the kidneys, flower: brewed in wine to enhance the fragrance
*Anredera cordifolia* (Tenore) Steenis	0.56	HRC1022	HL, YS, YZ		Neighborhood	Basellaceae	Liana	Forage, vegetable	Leaf	Raw or boiled as forage; eaten after boiled
*Broussonetia papyrifera* (Linnaeus) L’Heritier ex Ventenat	0.52	HRC978	HL, YS, DN, YZ	ba jie	Primary species, wild	Moraceae	Tree	Forage	Tender leaf	Cut, raw or boiled as forage
*Cinnamomum camphora* (L.) Presl	0.52	HRC1148	YS, DN, YZ	wo gu	Government	Lauraceae	Tree	Ornamental		
*Leucocasia gigantea* (Blume) Schott	0.52	HRC941	HL, YS, DN, YZ	wo bie	Wild, neighborhood	Araceae	Herb	Vegetable, forage	Petiole	Stir-fried after boiling as food; cut and boiled as forage
*Solanum lycopersicum* L	0.47	HRC1020	HL, YS, YZ	bi gua	Market, self-preservation	Solanaceae	Herb	Vegetable, medicine	Fruit, leaf	Eaten raw or stir-fried; making dipping sauce; mash and apply topically to treat burns or snakebites
*Ficus microcarpa* L	0.46	HRC1152	YS, DN		Market	Moraceae	Tree	Ornamental		
*Mentha canadensis* Linnaeus	0.46	HRC942	HL, YS, DN, YZ	yie wo	Neighborhood	Lamiaceae	Herb	Spice, medicine	Tender leaf	Making dipping sauces or as a spice; Chew fresh leaf for sore gums
*Zanthoxylum armatum* DC	0.46	HRC932	HL, YS, YZ	zu ya	Primary species, wild	Rutaceae	Tree	Vegetable, spice, medicine, veterinary drug	Leaf, fruit, branch	Leaf: eaten after boiled; externally used for snakebite; fruit: stir-fried or making dipping sauces, chewed for sore gums; branch: smash with rice for animal plague
*Perilla frutescens* (L.) Britt	0.45	HRC707	HL, DN, YS, YZ	deng huo jie	Self-preservation, neighborhood	Lamiaceae	Herb	Medicine, spice	Leaf	Steamed with eggs to treat diarrhea; making dipping sauce
*Castanea mollissima* Blume	0.42	HRC684	HL, DN, YZ	bi yi	Neighborhood, self-preservation	Fagaceae	Tree	Medicine, fruit	Fruit shell, fruit	Fruit shell: medicinal bath to treat itching; fruit: eaten after cooked
*Bougainvillea glabra* Choisy	0.41	HRC1106	YS, DN, HL		Market	Nyctaginaceae	Liana	Ornamental		
*Raphanus sativus* L	0.41	HRC1048	HL, YS, YZ	guo bo a	Market	Brassicaceae	Herb	Vegetable, forage	Root tuber, leaf	Root tuber: eaten freshly or making "sweet water", leaf: eaten after boiled; whole plant: raw or boiled as forage
*Zoysia japonica* Steud	0.41	HRC1151	YS, DN		Market	Poaceae	Herb	Ornamental		
*Beta vulgaris* var. *cicla* L	0.38	HRC1061	HL, YS, YZ	ya she	Market	Amaranthaceae	Herb	Vegetable, forage	Leaf	Eaten after boiled
*Lactuca sativa* var. *ramosa* Hort	0.36	HRC1034	HL, YZ		Market	Asteraceae	Herb	Vegetable	Leaf	Eaten after boiled
*Prunus salicina* Lindl	0.36	HRC1003	HL, YS, DN, YZ	pi	Neighborhood	Polygonaceae	Tree	Fruit	Fruit	Eaten after cooked
*Strobilanthes cusia* (Nees) Kuntze	0.36	HRC72	HL, YS, YZ	yin zhei	Neighborhood	Acanthaceae	Herb	Dye, veterinary drug	Branch	Fermented and made into indigo mud for dye or sell; smash with wine, salt, mud and *Gynura japonica* leaf, externally applied to treat pig wound
*Hibiscus syriacus* L	0.35	HRC950	HL, YS, YZ	wo wai	Neighborhood	Malvaceae	Shrub	Forage, vegetable	Tender branch, tender leaf	Tender branch: cut, raw or boiled as forage; tender leaf: eaten after boiled
*Pisum sativum* L	0.33	HRC1040	HL, YS, YZ	da a lai	Market, self-preservation	Fabaceae	Herb	Vegetable	Fruit, tender leaf	Eaten after boiled
*Ailanthus vilmoriniana* Dode	0.32	HRC647	HL, YS, DN, YZ	gu zhou	Neighborhood, self-preservation	Simaroubaceae	Tree	Resist agent, making ox horn	Resin	Reserving agent: before the white cotton cloth is ready to dye blue indigo, paint the cotton cloth with sticky paste(resin) to prevent the part from being dyed, dry the cloth, then recycle the resin for the following year; ox horn: after boiling, mixed with the reticular fiber at the base of the palm petiole, cooled quickly and glued to the horn to make a loudspeaker tube
*Allium hookeri* Thwaites	0.32	HRC1023	HL, YS, DN, YZ	ya ma li	Neighborhood	Amaryllidaceae	Herb	Vegetable	Leaf	Eaten after boiled
*Alpinia japonica* (Thunb.) Miq	0.32	HRC1139	YS, YZ	a giang zuo	Neighborhood	Zingiberaceae	Herb	Spice	Leaf	Add some leaves when cooking tofu
*Cichorium intybus* L	0.32	HRC1054	HL, YZ		Market	Asteraceae	Herb	Vegetable	Leaf	Eaten after boiled
*Cymbopogon citratus* (D. C.) Stapf	0.31	HRC1039	HL, YS, DN, YZ		Neighborhood	Poaceae	Herb	Spice, medicine	Leaf	Cook with fish or make dipping sauce; medicinal bath to relieve the heat; decoction with brown sugar to treat colds in children
*Pinus massoniana* Lamb	0.31	HRC51	DN	nong gua	Primary species	Pinaceae	Tree	Herbal tea, construction, ornamental	Pollen, stem	Pollen: make tea, it has the pine fragrance
*Canna indica* 'Edulis'	0.29	HRC106	HL, YZ	wu sao	Self-preservation, neighborhood	Cannaceae	Herb	Forage	Whole plant	Cut, raw or boiled as forage
*Peristrophe japonica* (Thunb.) Bremek	0.28	HRC581	YZ	zu miao	Neighborhood	Acanthaceae	Herb	Treating Gudu, medicine	Whole plant	Soaked in wine or decoction can treat stomachache or relieve Gudu
*Allium fistulosum* L	0.27	HRC1049	HL, YS, YZ	wo mie zha	Self-preservation	Amaryllidaceae	Herb	Spice	Leaf	Making dipping sauce
*Coix lacryma-jobi* L	0.27	HRC1159	YS, HL		Market, neighborhood	Poaceae	Herb	Making jewelry	Seed	String seeds to make necklaces or bracelets
*Citrus maxima* (Burm.) Merr	0.26	HRC1018	HL, YS, YZ	bi lia	Neighborhood, market	Rutaceae	Tree	Fruit, religious rite	Fruit, branch	Fruit: eaten directly after ripening; branch: hang on the door to ward off evil spirits
*Prunus persica* L	0.25	HRC479	HL, YS, DN, YZ	bi sua	Neighborhood, market	Rosaceae	Tree	Religious rite, fruit	Fruit, branch	Fruit: eaten directly after ripening; branch: hung on the door to ward off evil spirits or for religious rites
*Cucumis sativus* L	0.23	HRC336	HL, YS, DN, YZ	gong ga	Self-preservation	Cucurbitaceae	Herb	Medicine, vegetable	Tender leaf, fruit	Tender leaf: decoction for diarrhea; fruit: vegetable
*Pleioblastus amarus* (Keng) Keng f	0.22	HRC1115	HL, YS, DN, YZ	a zei	Neighborhood	Poaceae	Herb	Vegetable, construction, religious rite	Stem, shoot	Shoot: stir-fried after boiling or after soaking in water; old stem: weaving
*Bambusa vulgaris* f. *vittata* (Riviere & C. Riviere) T. P. Yi	0.21	HRC1132	YS, DN		Government	Poaceae	Tree	Ornamental		
*Bambusa ventricosa* McClure	0.2	HRC1154	DN		Government	Poaceae	Herb	Ornamental		
*Piper sarmentosum* Roxb	0.2	HRC1114	YS		Wild, neighborhood	Piperaceae	Herb	Spice	Leaf	Stir-fried with meat or making dipping sauce
*Vigna umbellata* (Thunb.) Ohwi et Ohashi	0.2	HRC1099	YS, YZ	da mian	Self-preservation, neighborhood	Fabaceae	Herb	Vegetable	Seed	Eaten after boiled
*Pyrus pyrifolia* (Burm. F.) Nakai	0.18	HRC1044	HL, YS, YZ	bi gu	Neighborhood	Rosaceae	Tree	Fruit	Fruit	Fruit: eaten after cooked
*Vigna unguiculata* (L.) Walp	0.18	HRC1025	HL, DN, YZ	da(dao)	Self-preservation	Fabaceae	Liana	Vegetable	Fruit	Stir-fried
*Opuntia dillenii* (Ker Gawl.) Haw	0.17	HRC325	HL, YS, YZ	zu ya niu	Market	Cactaceae	Shrub	Medicine, veterinary drug	Leaf	Mashed and external applied to treat inflammation, swelling, burn and scald; mashed and mixed into feed to treat animal plague
*Bambusa eutuldoides* McClure	0.16	HRC1180	YZ, HL		Neighborhood	Poaceae	Herb	Construction, religious rite	Stem	Bamboo weaving, or used as drum stick during religious rites
*Coriandrum sativum* L	0.16	HRC1157	DN, YZ		Market	Apiaceae	Herb	Spice	Leaf	Making dipping sauce
*Jasminum nudiflorum* Lindl	0.16	HRC1100	YS		Market	Oleaceae	Shrub	Ornamental		
*Musa basjoo* Sieb. et Zucc	0.16	HRC1036	HL, YS, YZ	wu sao	Neighborhood	Musaceae	Herb	Fruit, brewing, forage	Fruit, stem	Fruit: eaten directly after ripening; stem: chopped, mixed with yeast, sealed and fermented and distilled for wine; the lees can be forage
*Phytolacca acinosa* Roxb	0.16	HRC830	YS	zu ziong	Wild	Phytolaccaceae	Herb	Forage, medicine	Tender branch, root	Cut, raw or boiled as forage; root: stewed with meat for nourishing
*Rohdea japonica* (Thunb.) Roth	0.16	HRC1019	HL, YS, YZ	jio bie nong	Neighborhood	Asparagaceae	Herb	Medicine	Leaf	Mashed and steamed with pig liver, taken orally to treat Gudu, stomach pain, angina
*Senna surattensis* (N. L. Burman) H. S. Irwin & Barneby	0.16	HRC1166	YS		Government	Fabaceae	Shrub	Ornamental		
*Amorphophallus konjac* K. Koch	0.15	HRC943	HL, YS, YZ	gei niao ge bie	Self-preservation, neighborhood	Araceae	Herb	Vegetable	Stem tuber	Mash it into powder and add gypsum for precipitation to make konjac tofu
*Brassica juncea* (Linnaeus) Czernajew	0.15	HRC1175	YS, HL, YZ	wo bie ou	Market	Brassicaceae	Herb	Vegetable	Leaf	Making soup
*Lablab purpureus* (L.) Sweet	0.15	HRC403	HL, YZ	da zuo a	Market, self-preservation	Fabaceae	Liana	Vegetable	Fruit	Making soup
*Morella rubra* Lour	0.15	HRC1031	HL, YS, DN, YZ	ge bou	Primary species	Myricaceae	Tree	Fruit	Fruit	Fruit: eaten directly after ripening or soaked in wine
*Rhamnus utilis* Decne	0.15	HRC193	YS	nong ya	Primary species	Rhamnaceae	Shrub	Dye	Old stem	Boiled with water, mixed other dye plants for a yellow color
*Dioscorea persimilis* Prain et Burkill	0.14	HRC985	YS	du zai	Wild	Dioscoreaceae	Liana	Staple food, vegetable	Stem tuber	Peeled and stir-fried as food, or mashed with water before boiling
*Disporopsis pernyi* (Hua) Diels	0.14	HRC616	HL, YZ	zu nie	Neighborhood	Asparagaceae	Herb	Beauty, treating gudu	Whole plant	Medicinal bath for skin lightening; decoction for treating Gudu
*Nicotiana tabacum* L	0.14	HRC1087	YS, YZ	ran	Market	Solanaceae	Herb	Cigarette	Leaf	Dried and shredded, rolled into cigarettes
*Persicaria runcinata* var. *sinensis* (Hemsl.) Bo Li	0.14	HRC330	YZ	jio bao feng	Neighborhood	Polygonaceae	Herb	Medicine	Whole plant	Mashed and external applied to treat bruise and snakebite; cooked with chicken for postpartum recovery, or to treat infertility
*Rosa chinensis* Jacq	0.14	HRC1158	YS, YZ		Government	Rosaceae	Shrub	Ornamental		
*Bambusa chungii* McClure	0.13	HRC1032	HL	a mo a	Neighborhood	Poaceae	Herb	Vegetable, construction, religious rite	Tender shoot, old stem	The tender shoot is edible; the old stem can be used for weaving
*Cuphea hyssopifolia* Kunth	0.13	HRC1145	YS		Market	Lythraceae	Herb	Ornamental		
*Hovenia acerba* Lindl	0.13	HRC1169	HL, YS, YZ	bi zha	Neighborhood	Rhamnaceae	Tree	Ornamental, fruit	Fruit	Fruit: eaten directly after ripening or soaked in wine
*Maclura cochinchinensis* (Loureiro) Corner	0.13	HRC982	HL	ya su	Primary species	Moraceae	Shrub	Forage, fence	Tender leaf	Cut, raw or boiled as forage; making fence
*Platyosprion platycarpum* (Maxim.) Maxim	0.13	HRC945	HL	lan dian mu	Primary species	Fabaceae	Tree	Ornamental, sacred tree, fertilizer	Leaf	Leaf: fermented as fertilizer
*Podocarpus macrophyllus* (Thunb.) Sweet	0.13	HRC1155	YS, DN	bo hui	Government	Podocarpaceae	Tree	Ornamental		
*Acorus tatarinowii* Schott	0.12	HRC938	HL, YS, YZ		Neighborhood, wild	Acoraceae	Herb	Spice	Tender leaf	Stir-fried with meat or making dipping sauce
*Brassica oleracea* var. *capitata* Linnaeus	0.12	HRC1033	HL, YS, YZ		Market	Brassicaceae	Herb	Vegetable	Leaf	Eaten after stir-fried
*Clausena lansium* (Lour.) Skeels	0.12	HRC1178	YZ		Neighborhood	Rutaceae	Tree	Fruit	Fruit	Fruit: eaten directly after ripening or making pickle
*Clerodendrum bungei* Steud	0.12	HRC811	YS	wa sou	Wild	Lamiaceae	Shrub	Veterinary drug, medicine	Whole plant, branch, root	Whole plant: detection orally taken by animal to treat animal plague; branch: medicinal bath to treat hemorrhoids; root: medicinal bath to treat snakebite; leaf: external rubbed to relieve itching; root: decoction and orally taken to treat hypertension, sinusitis, rectal cancer
*Ficus altissima* Blume	0.12	HRC1082	YS		Market	Moraceae	Tree	Ornamental, medicine	Leaf	Smash and use externally to treat a bone fracture
*Juglans regia* L	0.12	HRC701	HL, YS, YZ		Government	Juglandaceae	Tree	Medicine, nut	Leaf, fruit	Leaf: medicinal bath for itching; fruit: eaten directly after ripening
*Ligustrum sinense* Lour	0.12	HRC1167	YS, HL, YZ		Primary species	Oleaceae	Shrub	Ornamental, fence		
*Lycium chinense* Miller	0.12	HRC944	HL, YZ		Neighborhood	Solanaceae	Shrub	Vegetable, forage	Tender leaf	Eaten after boiled; cut, raw or boiled as forage
*Melia azedarach* L	0.12	HRC1105	YS, YZ		Government	Meliaceae	Tree	Ornamental		
*Parthenocissus quinquefolia* (L.) Planch	0.12	HRC1163	YS		Market	Vitaceae	Liana	Ornamental		
*Prunus serrulata* var. *lannesiana* (Carri.) Makino	0.12	HRC1156	DN		Government	Rosaceae	Herb	Ornamental		
*Rumex nepalensis* Sprengel	0.12	HRC783	YZ		Neighborhood	Polygonaceae	Herb	Veterinary drug, medicine	Leaf, root tuber, whole plant	Leaf: mashed and external applied to treat cattle fall injury; whole plant: mashed and external applied to treat mumps; root tuber: decoction for sore throat
*Vitex negundo* L	0.12	HRC606	HL		Wild	Lamiaceae	Shrub	Treating gudu, medicine	Stem	Decoction or soaked in wine, orally taken to treat bruise, dysentery, dermatophytosis and Gudu
*Vitis vinifera* L	0.12	HRC1075	YS, YZ	bi gai	Market, neighborhood	Vitaceae	Liana	Fruit	Fruit, brewing	Fruit: eaten directly after ripening or making fruit wine
*Colocasia esculenta* (L.) Schott	0.11	HRC1176	YS, YZ	wo niang e	Self-preservation, neighborhood, market	Araceae	Herb	Vegetable, forage	Stem tuber, petiole, whole plant	Stem tuber: eaten after boiled, petiole: making pickle, then stir-fried or made into soup; the whole plant: cooked to make forage
*Cunninghamia lanceolata* (Lamb.) Hook	0.11	HRC696	HL, DN, YZ	luo ji	Market	Cupressaceae	Tree	Medicine, construction, religious rite	Leaf, stem	Leaf: mashed and external applied to treat snakebite; old stem: used for construction, coffin and component of bronze drum
*Euonymus japonicus* Thunb	0.11	HRC1073	YS		Market	Celastraceae	Shrub	Ornamental		
*Luffa aegyptiaca* Miller	0.11	HRC1057	HL, YS, YZ	guo bo a	Self-preservation, neighborhood	Cucurbitaceae	Liana	Vegetable, dish-washing	Fruit, tender shoot, loofah	Fruit, tender shoot: boiled with water before eaten; loofah can be used for dish-washing
*Toona sinensis* (A. Juss.) Roem	0.11	HRC981	HL, YS, DN, YZ	ya you	Neighborhood	Meliaceae	Tree	Vegetable, dye	Tender leaf, bark	Tender leaf: blenched and then stir-fried with meat or eggs before eaten; bark: decocted until the water turned red, soaked the cotton in the decoction, then turned black after dried
*Cupressus funebris* Endl	0.1	HRC1153	DN, YZ	nong gua	Market, neighborhood	Cupressaceae	Tree	Ornamental, religious rite	Branch	Branch: hung the door to ward off evil spirits and for a religious rite
*Dioscorea cirrhosa* Lour	0.1	HRC98	HL, YZ	nei re	Wild	Dioscoreaceae	Liana	Dye	Stem tuber	Sliced up and decocted, soak cloth into the decoction, then sun-dried, repeated 3–4 times, to fix and brighten the cotton cloth
*Firmiana simplex* (Linnaeus) W. Wight	0.1	HRC639	HL, YZ	jiu ceng pi	Government	Malvaceae	Tree	Making rope	Bark	Peeled and soaked in water for about a week, and then take the inner layer of white skin to dry to make rope
*Hippeastrum vittatum* (L'Her.) Herb	0.1	HRC1108	YS, YZ		Neighborhood, market	Amaryllidaceae	Herb	Ornamental		
*Lactuca sativa* var. *angustata* Irish ex Bremer	0.1	HRC1021	HL, DN, YZ		Market	Asteraceae	Herb	Vegetable	Leaf	Eaten after boiled
*Platycladus orientalis* (L.) Franco	0.1	HRC980	HL, YZ	lu ya(nong gua)	Neighborhood	Cupressaceae	Tree	Religious rite, sacred tree, construction	Old stem	The old stem can be used to make looms
*Tetradium ruticarpum* (A. Jussieu) T. G. Hartley	0.1	HRC568	YZ	mi la	Wild	Rutaceae	Tree	Medicine, veterinary drug	Branch, fruit	Branch: decocted for internal administration to treat animal plague, fruit: orally taken with water can aid digestion
*Jacaranda mimosifolia* D. Don	0.09	HRC1160	YS		Market	Bignoniaceae	Tree	Ornamental		
*Lagerstroemia indica* L	0.09	HRC1027	HL, YZ		Market	Lythraceae	Tree	Ornamental		
*Aglaonema modestum* Schott ex Engl	0.08	HRC1172	YS		Market	Araceae	Herb	Ornamental		
*Asparagus cochinchinensis* (Lour.) Merr	0.08	HRC144	YS	qi jie mei	Wild	Asparagaceae	Herb	Medicine	Root tuber	Cooked with porridge; soaked in wine, and orally taken to treat impotence
*Canna indica* L	0.08	HRC788	HL, YZ		Market	Cannaceae	Herb	Medicine	Whole plant	Minced and cooked with meat
*Curcuma longa* L	0.08	HRC552	YZ	gang	Wild	Zingiberaceae	Herb	Medicine, dye	Root tuber	Mashed and external applied to treat a bruise, hepatitis, or for wound healing; meshed and soaked in water to dye white silk for the yellow color
*Eucommia ulmoides* Oliver	0.08	HRC353	YZ		Government	Eucommiaceae	Tree	Medicine	Bark	Mashed and external applied to treat bruise and swelling; decoction or stewed with meat for nourishing
*Punica granatum* L	0.08	HRC1037	HL, YS, YZ		Market	Lythraceae	Tree	Fruit, ornamental	Fruit	Fruit: eaten directly after ripening
*Stephania dielsiana* Y. C. Wu	0.08	HRC1070	YS	zhun zu wo	Wild	Menispermaceae	Liana	Medicine	Stem tuber	Ground into powder and orally taken with water to treat indigestion and diarrhea
*Torricellia tiliifolia* DC	0.08	HRC741	HL, YZ	nong wo bo	Neighborhood	Toricelliaceae	Tree	Medicine, veterinary drug, fertilizer	Whole plant, leaf	Whole plant: mashed and external applied to treat bone fracture; leaf: medicinal bath to treat limb ache; leaf: mashed and external applied to treat animal wounds; leaf: fermented to be fertilizer
*Trachycarpus fortunei* (Hook.) H. Wendl	0.08	HRC946	HL, YZ	ye li	Neighborhood	Arecaceae	Tree	Ornamental, construction	Whole plant, reticular fibers at the base of petiole	The whole plant for ornamental; reticular fibers at the base of the petiole are used for making broom or ox horn
*Tradescantia zebrina* Bosse	0.07	HRC1118	YS		Neighborhood	Commelinaceae	Herb	Ornamental, medicine	Whole plant	Soaked in wine for oral administration for nourishing
*Acorus gramineus* Soland	0.06	HRC1090	YS		Wild	Acoraceae	Herb	Spice	Leaf	Making dipping sauce
*Bauhinia brachycarpa* Wall. ex Benth	0.06	HRC1010	HL		Government	Fabaceae	Shrub	Fence, ornamental		Making fence
*Boehmeria nivea* (L.) Gaudich	0.06	HRC1030	HL, YZ	wo gu	Primary species	Urticaceae	Shrub	Forage, staple food	Leaf, root stem	Leaf: cut, raw or boiled as forage; root stem: mashed and cooked as food
*Celtis biondii* Pamp	0.06	HRC1008	HL		Primary species	Cannabaceae	Tree	Ornamental, sacred tree		
*Cinnamomum burmanni*	0.06	HRC1177	YZ		Government	Lauraceae	Tree	Ornamental		
*Citrus reticulata* Blanco	0.06	HRC1171	YS, HL, YZ	bi li	Market	Rutaceae	Tree	Fruit	Fruit	
*Diospyros kaki* Thunb	0.06	HRC1012	HL, YZ	pi bei	Market	Ebenaceae	Tree	Fruit	Fruit	Fruit: eaten directly after ripening
*Ginkgo biloba* L	0.06	HRC1014	HL, YZ		Government	Ginkgoaceae	Tree	Ornamental		
*Keteleeria davidiana* var. *calcarea* (W. C. Cheng & L. K. Fu) Silba	0.06	HRC1016	HL		Primary species	Pinaceae	Tree	Sacred tree, ornamental		
*Ligustrum lucidum* Ait	0.06	HRC1029	HL, YS, YZ		Government	Oleaceae	Tree	Ornamental, custom plant, medicine	Whole plant, branch, fruit	Branch: during the Spring Festival, branches will be burned to bake clothes to ward off bad luck; fruit is traditional Chinese medicine. People would buy it in the past
*Murraya exotica* L. Mant	0.06	HRC1076	YS		Wild	Rutaceae	Tree	Ornamental		
*Pennisetum purpureum* Schum	0.06	HRC835	HL, YS, YZ		Market	Poaceae	Herb	Forage	Tender branch	Cut, raw or boiled as forage
*Pistacia chinensis* Bunge	0.06	HRC1017	HL		Primary species	Anacardiaceae	Tree	Sacred tree, ornamental		
*Ricinus communis* L	0.06	HRC422	YS, YZ	de he pu	Neighborhood, wild	Euphorbiaceae	Herb	Medicine	Leaf	Mashed and external applied to treat paralysis and rectocele
*Alangium chinense* (Lour.) Harms	0.05	HRC1009	HL	bai xin tiao	Primary species	Cornaceae	Tree	Fence		Fence
*Basella alba* L	0.05	HRC1112	YS		Market	Basellaceae	Herb	Vegetable	Tender branch	Eaten after boiled
*Dioscorea subcalva* Prain et Burkill	0.05	HRC490	YS	du lu	Wild	Dioscoreaceae	Liana	Construction, staple food	Stem tuber	Mashed, add water and cook with cotton thread, constantly stirring to strengthen the toughness of the cotton thread; mashed and cooked as a staple food
*Eleutherococcus nodiflorus* (Dunn) S. Y. Hu	0.05	HRC87	HL, YZ	zu ji bi	Neighborhood	Araliaceae	Shrub	Medicine	Root	Soaked in wine and administrated orally to treat injury
*Euphorbia royleana* Boiss	0.05	HRC1081	YS		Market	Euphorbiaceae	Shrub	Ornamental		
*Hydrocotyle verticillata* Thunb	0.05	HRC1111	YS		Market	Araliaceae	Herb	Ornamental		
*Lantana camara* L	0.05	HRC1124	YS		Market	Verbenaceae	Shrub	Ornamental		
*Morus australis* Poir	0.05	HRC1078	YS		Neighborhood	Moraceae	Shrub	Forage, fruit	Leaf, fruit	Leaf: used as feed for silkworms or pigs; fruit: eaten directly
*Parthenocissus dalzielii* Gagnep	0.05	HRC1164	YS		Market	Vitaceae	Liana	Ornamental		
*Pterolobium punctatum* Hemsl	0.05	HRC1011	HL		Primary species	Fabaceae	Liana	Fence, medicine, religious rite	Branch	Tender leaf: mashed and external applied to treat snakebite; decocted for external washing to treat eye pain; branch: used to expel the evil spirit or used as fence
*Salix babylonica* L	0.05	HRC1072	YS, YZ		Market	Salicaceae	Tree	Ornamental		
*Sechium edule* (Jacq.) Swartz	0.05	HRC1058	HL, YS, YZ		Self-preservation, neighborhood	Cucurbitaceae	Liana	Vegetable, forage	Fruit	Fruit: stir-fried for food; cut into pieces and boiled as forage
*Sorghum bicolor* (L.) Moench	0.05	HRC1041	HL, YS, YZ	a yong	Self-preservation	Poaceae	Herb	Forage, food	Fruit	Fruit: fermented for wine, tender leaf: freshly or boiled as forage
*Zingiber mioga* (Thunb.) Rosc	0.05	HRC890	YZ		Wild	Zingiberaceae	Herb	Medicine, vegetable	Whole plant, tender inflorescence	Whole plant: medicinal bath to activate the nervous system and to treat paralysis; tender inflorescence: blenched and stir-fried before eaten
*Caryota maxima* Blume ex Martius	0.04	HRC1097	YS		Primary species	Arecaceae	Tree	Ornamental		
*Hymenocallis littoralis* (Jacq.) Salisb	0.04	HRC767	YS, YZ	bie wen	Market, neighborhood	Amaryllidaceae	Herb	Medicine, ornamental	Whole plant	Medicinal bath to treat arthritis and bone fracture, decocted for internal administration to treat pneumonia
*Ligularia hodgsonii* Hook	0.04	HRC363	YZ		Neighborhood	Asteraceae	Herb	Medicine	Whole plant	Decocted for internal administration to treat cough, tuberculosis
*Photinia serratifolia* (Desfontaines) Kalkman	0.04	HRC1007	HL		Primary species	Rosaceae	小tree	Ornamental, sacred tree		
*Phrynium rheedei* Suresh & Nicolson	0.04	HRC1060	HL, YZ		Wild, neighborhood	Marantaceae	Herb	Rapping zongzi	Leaf	Rapping zongzi
*Alocasia cucullata* (Lour.) Schott	0.03	HRC640	HL, YS	wu	Neighborhood	Araceae	Herb	Medicine	Stem tuber	Treat toothache: shredded and roasted until half-cooked in the fire and put it in the place of toothache for a while; medicinal bath to treat fever
*Alpinia zerumbet* 'Variegata'	0.03	HRC1165	YS		Market	Zingiberaceae	Herb	Ornamental		
*Arundo donax* L	0.03	HRC383	YZ	yi guo	Primary species	Poaceae	Herb	Medicine	Stem	Decocted for internal administration to treat mad dog bites; medicinal bath for postpartum recovery
*Belamcanda chinensis* (L.) Redouté	0.03	HRC465	YZ	nong bie	Neighborhood	Iridaceae	Herb	Medicine	Whole plant	Decocted for internal administration to treat a bruise, and to activate blood and remove stasis
*Buxus bodinieri* Lévl	0.03	HRC1144	YS		Market	Buxaceae	Shrub	Ornamental		
*Buxus sinica* (Rehd. et Wils.) Cheng	0.03	HRC1162	YS, YZ		Market	Buxaceae	Shrub	Ornamental		
*Chlorophytum comosum* (Thunb.) Baker	0.03	HRC1141	YS, YZ		Market	Asparagaceae	Herb	Ornamental		
*Citrus reticulata* 'Shatang'	0.03	HRC1084	YS		Market	Rutaceae	Tree	Fruit	Fruit	Eaten freshly as fruit
*Cyperus cyperoides* (L.) Kuntze	0.03	HRC1168	YS		Market	Cyperaceae	Herb	Ornamental		
*Gladiolus gandavensis* Van Houtte	0.03	HRC355	HL, YZ	suan pan guo	Market, neighborhood	Iridaceae	Herb	Medicine	Whole plant	Decocted for internal administration to treat sore throat, cough
*Gynura bicolor* (Willd.) DC	0.03	HRC939	HL, YS		Neighborhood	Asteraceae	Herb	Vegetable, forage	Tender leaf	Eaten after boiled; chopped, boiled or freshly fed to pigs
*Hylocereus undatus* (Haw.) Britt. et Rose	0.03	HRC964	HL		Market	Cactaceae	Shrub	Forage	Flower	Cut and boiled as forage
*Impatiens balsamina* L	0.03	HRC1109	YS	zu nao ai mi	Wild, self-preservation	Balsaminaceae	Herb	Ornamental, medicine	Seed	Orally taken to treat postpartum placenta residue
*Liquidambar formosana* Hance	0.03	HRC573	HL, DN, YZ	yin mei	Primary species	Altingiaceae	Tree	Medicine, veterinary drug, sacred tree	Leaf	Mashed, soaked in water and then fed the cattle to treat diarrhea; decocted for internal administration to treat stomachache, and diarrhea; medicinal bath to treat hemiplegia
*Nephrolepis cordifolia* (Linnaeus) C. Presl	0.03	HRC1161	YS	jie guo	Wild	Nephrolepidaceae	Herb	Ornamental		
*Polygonatum cyrtonema* Hua	0.03	HRC611	HL	zu suo xi	Wild	Asparagaceae	Herb	Medicine	Stem tuber	Stew with pig feet for nourishing
*Pyracantha fortuneana* (Maxim.) Li	0.03	HRC984	DN	gong jie	Wild	Rosaceae	Shrub	Fruit, ornamental	Fruit	Fruit: eaten directly after ripening
*Rhus chinensis* Mill	0.03	HRC993	YS	dang ji bu	Primary species	Anacardiaceae	Tree	Making kansui, fence	Fruit	Soaking in water to make Kansui; making tofu; making fence
*Stephania kwangsiensis* Lo	0.03	HRC782	YS, YZ	zu lu	Wild	Menispermaceae	Liana	Medicine, veterinary drug	Stem tuber	Mashed and decocted for internal administration to treat pig and cow plague and flatulence; mashed and external applied to treat bruise; decocted for internal administration to treat colorectal cancer and gastritis
*Tetrastigma hemsleyanum* Diels et Gilg	0.03	HRC1079	YS	ai zhe	Wild	Vitaceae	Liana	Ornamental, medicine	Whole plant	Decocted and taken orally to relieve Gudu
*Tradescantia pallida* (Rose) D. R. Hunt	0.03	HRC1117	YS, YZ		Neighborhood	Commelinaceae	Herb	Ornamental, medicine	Whole plant	External rub to treat vitiligo
*Artemisia indica* Willd	0.02	HRC1026	HL	wa huo	Primary species	Asteraceae	Herb	Vegetable, medicine	Tender leaf	Tender leaf: eaten after boiled; leaf: mashed and external applied to stop bleeding, decoction for dysentery
*Bambusa pervariabilis* McClure	0.02	HRC1130	YS		Government	Poaceae	Herb	Ornamental, construction, religious rite	Stem	Old stem: weaving or making drum sticks
*Coreopsis grandiflora* Hogg	0.02	HRC1142	YS		Market	Asteraceae	Herb	Ornamental		
*Curculigo capitulata* (Lour.) O. Kuntze	0.02	HRC1028	HL, YS, YZ	zu lie li	Wild	Hypoxidaceae	Herb	Veterinary drug	Leaf	Chopped and boiled to feed cattle to kill parasites
*Diospyros oleifera* Cheng	0.02	HRC1013	HL, YS, YZ	pi bei	Neighborhood	Ebenaceae	Tree	Fruit	Fruit	Eaten directly after ripening
*Eleutherococcus trifoliatus* (Linnaeus) S. Y. Hu	0.02	HRC642	HL		Neighborhood	Araliaceae	Shrub	Medicine	Root	Cooked with meat for nourishing; soaked in wine, and taken orally to treat a bone injury
*Gynura japonica* (Thunb.) Juel	0.02	HRC641	HL, YZ	zu wan cai	Neighborhood	Asteraceae	Herb	Vegetable, medicine, forage	Leaf	Boiled as a vegetable; mashed and external applied to treat a bruise
*Houttuynia cordata* Thunb	0.02	HRC1136	YS	ya zha	Wild	Saururaceae	Herb	Ornamental, vegetable, medicine	Tender leaf, whole plant	Tender leaf: stir-fried or made into a salad; whole plant: decocted for internal administration to treat jaundice, sore limbs; medicinal bath to treat edema
*Ilex kwangtungensis* Merr	0.02	HRC660	HL		Neighborhood	Aquifoliaceae	Tree	Dye	Leaf	Meshed and cooked with other dye plants in water to dye silk for a reddish-brown color
*Indocalamus longiauritus* Handel-Mazzetti	0.02	HRC1002	HL	nong jiu	Primary species	Poaceae	Herb	Rapping zongzi	Leaf	Rapping zongzi
*Juniperus chinensis* L	0.02	HRC1150	YS	nong guo a	Market	Cupressaceae	Tree	Ornamental		
*Momordica subangulata* Bl	0.02	HRC1088	YS		Neighborhood	Cucurbitaceae	Herb	Vegetable	Fruit	Eaten after boiled
*Mucuna birdwoodiana* Tutch	0.02	HRC306	HL		Wild	Fabaceae	Liana	Medicine, dye, religious rite	Old stem, root, tender branch	Old stem, root: medicinal bath to treat bruise and paralysis; tender branch: meshed and soaked in water until the water turns red, can make the indigo dye darker and fix the color; old stem: used as a drum stick
*Nerium oleander* L	0.02	HRC1103	YS		Government	Apocynaceae	Shrub	Ornamental		
*Phoebe bournei* (Hemsl.) Yang	0.02	HRC1173	DN		Government	Lauraceae	Tree	Ornamental, construction		
*Pistacia weinmanniifolia* J. Poisson ex Franchet	0.02	HRC1126	YS, YZ		Market, wild	Anacardiaceae	Tree	Ornamental		
*Rhapis humilis* Bl	0.02	HRC1174	YS, DN		Market	Arecaceae	Shrub	Ornamental		
*Saccharum officinarum* L	0.02	HRC1042	HL, DN, YZ		Neighborhood	Poaceae	Herb	Fruit	Stem	Peeled and eaten freshly
*Sambucus javanica* Blume	0.02	HRC631	HL, YS, YZ	nuo bo xi	Wild	Adoxaceae	Herb	Medicine	Leaf	Mashed and external applied to treat a bruise
*Smallanthus sonchifolius* (Poepp.) H.Rob	0.02	HRC1085	YS, YZ		Neighborhood	Asteraceae	Herb	Fruit	Stem tuber	Eaten directly
*Solanum pseudocapsicum* L	0.02	HRC1133	YS		Market	Solanaceae	Shrub	Ornamental		
*Viburnum odoratissimum* Ker.-Gawl	0.02	HRC1179	YZ, HL		Government	Adoxaceae	Tree	Ornamental, fence		
*Vitex quinata* (Lour.) Will	0.02	HRC605	HL	yi zhu nuo	Primary species	Lamiaceae	Tree	Treating gudu, medicine	Stem	Decocted or soaked in water for oral administration to treat bruise, dysentery, beriberi and to relieve Gudu
*Aconitum carmichaelii* Debeaux	0.01	HRC632	HL	zu nu	Neighborhood	Ranunculaceae	Herb	Medicine, veterinary drug	Root tuber	Soaked in wine and apply externally to treat bruise; mixed with feed to kill parasites and nourish livestock
*Asparagus lycopodineus* (Baker) Wang et Tang	0.01	HRC806	YS	qi jie mei	Wild	Asparagaceae	Herb	Medicine	Root tuber	Cook porridge with rice
*Bletilla striata* (Thunb. ex Murray) Rchb. F	0.01	HRC44	HL	zhe he	Wild	Orchidaceae	Herb	Beauty	Stem tuber	Decocted for internal administration for beauty
*Caesalpinia decapetala* (Roth) Alston	0.01	HRC1038	HL, DN, YZ		Primary species	Fabaceae	Liana	Fence		fence
*Cestrum nocturnum* L	0.01	HRC1121	YS		Market	Solanaceae	Shrub	Ornamental		
*Cycas revoluta* Thunb	0.01	HRC1129	YS		Government	Cycadaceae	Liana	Ornamental		
*Dendrobium officinale* Kimura et Migo	0.01	HRC1063	HL		Wild	Orchidaceae	Herb	Medicine	Whole plant	Mostly sold to other places
*Diospyros kaki* var. *silvestris* Makino	0.01	HRC996	HL	jin bei	Primary species	Ebenaceae	Tree	Fruit	Fruit	Fruit: eaten directly after ripening
*Edgeworthia chrysantha* Lindl	0.01	HRC635	HL		Neighborhood	Thymelaeaceae	Shrub	Medicine	Whole plant	Mashed and external applied to treat a bone fracture
*Euphorbia lathyris* Linnaeus	0.01	HRC457	YZ		Neighborhood	Euphorbiaceae	Herb	Medicine	Fruit	Orally taken to treat constipation (small amount, slightly toxic)
*Fagraea ceilanica* Thunb	0.01	HRC1127	YS, DN		Market	Gentianaceae	Tree	Ornamental		
*Lilium brownii* F. E. Brown ex Miellez	0.01	HRC576	YS		Wild	Liliaceae	Herb	Vegetable	Bulb, leaf	Eaten after boiled
*Lilium brownii* var. viridulum Baker	0.01	HRC983	YS	gai	Wild	Liliaceae	Herb	Vegetable, ornamental	Bulb	Stir-fried or stewed with meat
*Nandina domestica* Thunb	0.01	HRC579	HL, YZ	nong gei li	Primary species	Berberidaceae	Shrub	Treating Gudu, medicine, fence	Stem	Soaked in wine or decocted, then taken orally to treat stomach pain, cold, cough and Gudu
*Phedimus aizoon* (Linnaeus) 't Hart	0.01	HRC1123	YS		Market	Crassulaceae	Herb	Ornamental		
*Prunus mume* Siebold & Zucc	0.01	HRC1170	YS	bi ma	Neighborhood	Rosaceae	Tree	Ornamental, fruit	Fruit	Fruit: eaten directly after ripening or making pickle
*Ruta graveolens* L	0.01	HRC1069	YS		Government	Rutaceae	Herb	Medicine	Whole plant	Mashed and external applied to stop bleeding, relieve pain and inflammation; decoction and taken orally to treat the urgency-frequency syndrome
*Solanum torvum* Swartz	0.01	HRC1147	YS		Primary species	Solanaceae	Shrub	Ornamental		

HL represents Huaili Village; YZ represents Yaozhai Village; YS represents Yaoshan Village, and DN represents Duonu Community

The order of plants follows by the RFC value and alphabetical order of Latin initials

Herbaceous plants (101 species; 47.42%) are the dominant components of Baiku Yao homegardens, followed by trees (61 species; 28.64%), shrubs (31 species; 14.55%), and lianas (20 species; 9.39%). The rich species diversity and multiple layers of planting are the characteristic features of Baiku Yao homegardens.

**Fig. 3:**
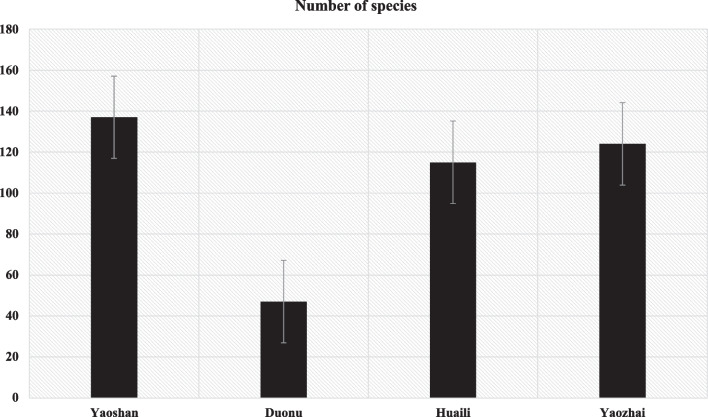
Comparison of species numbers among different villages

Among the four investigated villages, Yaoshan Village had the highest diversity of homegarden plants (137 species; 64.32%), followed by Yaozhai Village (124 species; 58.22%), Huaili Village (119 species; 53.99%) and Duonu Community (47 species; 22.07%). The statistics in Fig. 3 show that Baiku Yao residing in Yaoshan Village have well-developed diversity of homegarden plants during relocation. Unlike the traditional homegardens in ancient Baiku Yao villages, ornamental plants are the key components of homegardens in Yaoshan. With increasing local tourism, the demand for ornamental species has increased. The traditional ancient villages (Yaozhai and Huaili Village) of Baiku Yao mainly cultivate food plants and medicinal plants in their homegardens to sustain their daily livelihood. Moreover, these two ancient villages have not been exposed to tourism; therefore, they maintain only those species they require for their own consumption, leading to similar species numbers in their homegardens. In contrast, Duonu Community is one of the newly built relocated villages for Baiku Yao. Therefore, their homegardens are still in the initial development and have less species diversity. According to observations, in Duonu Community, local people mainly grow ornamental species such as *Podocarpus macrophyllus*, *Zoysia japonica*, *Bambusa ventricosa*, and *Bambusa* vulgaris 'Vittata'.

**Fig. 4:**
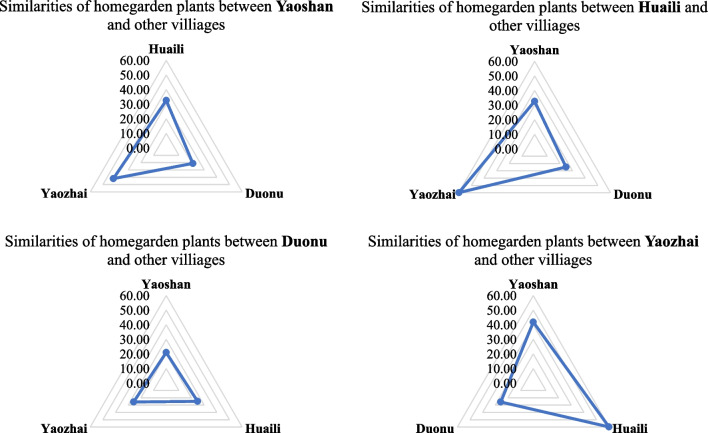
JI value of homegarden plants among four villages

In this study, the JI value is used to express the similarity of homegarden plants in four villages (Fig. 4); the higher the JI value is, the higher the similarity of homegarden plants between the two villages [5]. According to the calculation, Yaozhai Village and Huaili Village had the highest JI values (59.87%), reflecting the very high similarity of homegarden plants and indicating the frequent communication and exchange of traditional knowledge between these two ancient Baiku Yao villages. The similarity between Yaoshan and Yaozhai (41.85%) and Yaoshan and Huaili (32.64%) also seems relatively good. Compared to Duonu Community, Yaoshan villagers may communicate more with ancient villages and practice cultivating different homegarden plants even after relocation. However, the lower similarity of homegarden plants of Duonu Community with other villages, such as Yaozhai (25.74%), Huaili (24.81%), and Yaoshan (21.05%), could be linked to the limited period since their recent relocation. Most of the homegardens have not started cultivating crops or transplanted wild plants.

### Sources of homegarden plants

**Fig. 5:**
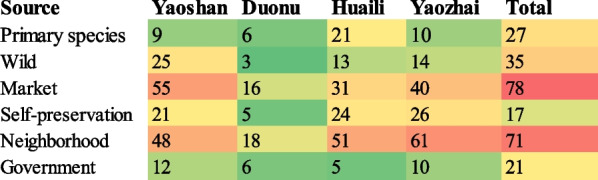
Heatmap of plant sources of homegarden plants in Baiku Yao villages

Homegarden plants of Baiku Yao normally come from various sources, such as (i) primary species (originally found in the locality even before any human interventions, 27 species), (ii) the wild environment (transplanted from the wild, 35 species) and (iii) neighborhood exchanges (71 species). Propagules (i.e., seeds and seedlings) of some species are purchased from the market (78 species), and some species are self-preserved (27 species) by Baiku Yao to maintain their homegardens (Fig. 5). In relocated villages, the local government also encourages them to grow ornamental species (21 species) and provides them with planting material to enhance their livelihood and decorate the community. Many homegarden plant provenances come from multiple sources, such as *Perilla frutescens*, *Agastache rugosa*, and *Zea mays*, from both markets and self-preservation; *Acorus tatarinowii* comes not only from the wild but also from neighbor sharing. According to the investigation, among all villages we visited, market purchases contributed the most to maintaining Baiku Yao homegardens (36.62%), followed by neighborhood sharing (33.33%), wild (16.43%), primary species (12.68%), government issuance (9.86%) and self-preserved species (7.98%).

According to Fig. 5, most of the homegarden plants in ancient villages were from neighbor sharing (61 species in Yaozhai and 51 species in Huaili), followed by purchasing from the market (40 species in Yaozhai, 31 species in Huaili) and self-preservation species (26 species in Yaozhai, 24 species in Huaili). However, in Yaoshan, an early relocated village, most homegarden plants were purchased from the market (55 species), followed by neighbor sharing (48 species). According to the interviews, Yaoshan villagers depend on exchange plants with other villages for complementary resources. However, in Duonu Community, which was recently relocated, homegarden plants mainly come from neighbor sharing (18 species) and market purchases (16 species).

The introduction of homegarden plants from neighbor-sharing accounts for a very high proportion in the four villages, indicating the presence of frequent internal communication in Baiku Yao village; they have a regular seed and seedling exchange network. Moreover, Baiku Yao has a high degree of recognition of food, culture, aesthetic appreciation, and consistent living habits. We also observed that a certain number of primary species are common in the homegardens of each study village, of which Huaili (21 species) is the highest, indicating worship and respect for nature among Baiku Yao, who make full use of the value of primary plants in homegarden design. As an ancient village, Huaili has well preserved and inherited the life concept of Baiku Yao.

### Analysis of the functions of homegarden plants

**Fig. 6:**
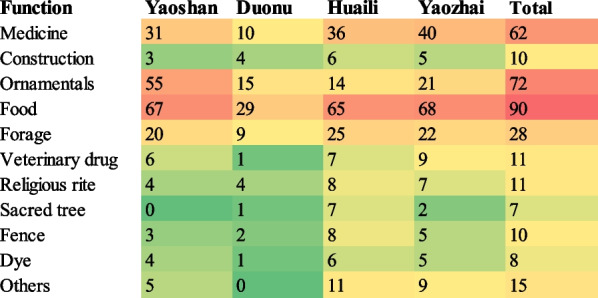
Heatmap of plant functions of homegarden plants in Baiku Yao villages

The homegarden plants of Baiku Yao have a wide range of uses. We classified these plants into 11 types (Fig. 6) based on their use value, including medicine, timber, ornamental, food, foraging, veterinary medicine, religious rites, sacred trees, fences, dyes, and others (cigarettes, beauty, rope, and treat Gudu, a poison produced by venomous insects or evil curses). Edible plants accounted for the majority (90 species), followed by ornamentals (72 species), medicine (62 species), forage (28 species), veterinary medicine (11 species), religious rituals (11 species), timber (10 species), fences (10 species), dyes (8 species), and sacred trees (7 species).

The number of edible plant species was high (above 50% of all plant species) in the homegarden of each village (Fig. 6), indicating that the homegardens of Baiku Yao are the primary source for providing food by cultivating crops, vegetables, and fruits.

Medicinal plants occupied the second position in the homegardens of different villages, i.e., Huaili (36 species, 30.25%), Yaozhai (40 species, 32.26%), Yaoshan (31 species, 22.63%), and Daonu (10 species, 21.3%). These findings indicate that Baiku Yao homegardens play an important role in community disease prevention and treatment, especially in Baiku Yao's ancient villages.

Approximately 15–20% of species represent forage plants in the homegardens of each village. The villagers have generally raised poultry and livestock since ancient times. Most Baiku Yao insists on using traditional wild vegetables or planting coarse grains in the feeding process instead of market feeds. The locals believe that the calories of commercial feed are too high, and the livestock is prone to internal fever and digestion problems [3]. Therefore, forage species are important components of homegardens in both traditional and relocated villages.

Ornamental plant diversity varies significantly among the studied villages. For example, Yaoshan Village has the highest number of ornamental plants (55 species; 40.15%), followed by Yaozhai (21 species, 16.94%), Duonu (15 species, 31.9%), and Huaili (14 species; 11.76%). The statistics of ornamental species indicate that Baiku Yao integrates various ornamental plants in constructing and designing homegardens; thus, they have a rich diversity of ornamental plants. Yaoshan Village has the most diverse ornamental plants because of local tourism development. In addition to the traditional ornamental plants the locals like, there are many landscape plants planned by the local government and tourism companies.

A comparison of homegarden plants among the four villages revealed that the distribution of plants utilized for veterinary medicine, religious rites, timber, fence, and dyes is much higher in the homegardens of ancient villages (Yaozhai and Huaili) compared to relocated villages (Yaoshan and Duonu). We found scarcely any sacred trees in Yaoshan Village, while Duonu Community homegardens had only one species of veterinary plant, one species of dye plant, and one species of the sacred tree. The comparison indicates that the traditional practice of homegarden plants in Baiku Yao ancient villages is higher and more comprehensive than in relocated villages. Because the relocated population may need to deal with a new natural resources composition or even face a different social environment, which leads to a changing livelihood or a different frequency of practice of traditional knowledge, relocation could be responsible for eroding some traditional knowledge. There are fewer types of homegarden plants in the Duonu Community than in other villages, meaning that establishing plant diversity in traditional homegardens requires a long-term process.

### The impact of the tourism industry on studied villages

Our results show that ancient Baiku Yao villages (Huaili and Yaozhai) have more diversity of homegarden plants, which may suggest that compared to relocated villages, traditional culture related to homegarden plants in the ancient villages has been affected less by the modernization or the development of tourist industry. Duonu Community has much less diversity of homegarden plants. After relocation, these people moved to a different area with different environmental conditions and topography compared to their native location. They may not have been able to find and cultivate the same species as they used to grow in their native locations, and some of the traditional knowledge could be lost with time, which is somehow reflected by the low species richness in their homegardens. In contrast, homegardens in Yaoshan Village have a high diversity of species, similar to the two ancient villages. The field visit showed that Yaoshan villagers preserved not only relevant traditional knowledge but also incorporated new species into their homegardens even after relocation. A higher proportion of homegarden plants in Yaoshan Village comes from the market because, after relocation, they had more opportunities to travel to different places due to convenient transportation facilities. Therefore, they incorporated many new species into their homegardens. Due to local tourism development, they also incorporated many horticultural species in their homegardens compared to ancient villages.

Tourism activities were introduced in Yaoshan Village in 2009 to generate employment for local people under the national poverty reduction program and present the Baiku Yao ethnic culture to others. The tourism company improved road connectivity and developed infrastructure facilities to attract tourists, thus increasing local employment for the Baiku Yao people. During the investigation, we observed that although Yaoshan Village had become a tourist destination, it still maintained and preserved many traditional practices for homegarden plants. These practices might help maintain their traditional knowledge during tourism development. According to the introduction from the tourism company staff, the company believes that tourism should focus on the folk culture of Baiku Yao rather than paying attention to and interfering in the development of regional economy. This is reflected by the rich species diversity they plant in their homegardens to sustain their daily requirements. Tourism companies provide good infrastructure (such as roads, tap water, electricity, and house repair), but they keep their best not to interfere with the routine life of Baiku Yao, such as growing vegetables, sericulture, keeping chickens, and pigs. Therefore, the concept of traditional cultural protection preserves not only local traditional knowledge but also gives local tourism more cultural connotation and experience. Consequently, many people are attracted by the local tourism and ecotourism, which ultimately drives the development of the regional economy.

### RFC value analysis of homegarden plants

The RFC values ranged from 0.01 to 0.93. The RFC value indicates the frequency of a specific species in the homegardens of Baiku Yao and reflects its importance in local daily life. Approximately 22 homegarden plants showed a high RFC value (> 0.5). Among them, *Zea mays* had the highest RFC value (0.93), followed by *Morus alba* (0.87), *Capsicum annuum* (0.87), *Eriobotrya japonica* (0.68), *Ipomoea batatas* (0.68), *Solanum melongena* (0.68), *Glycine max* (0.67), *Phaseolus vulgaris* (0.66), *Agastache rugosa* (0.65), *Cannabis sativa* (0.65), *Brassica rapa* var. *oleifera* (0.62), *Cucurbita moschata* (0.62), *Zingiber officinale* (0.6), *Amaranthus tricolor* (0.58), *Allium sativum* (0.58), *Osmanthus fragrans* (0.57), *Fagopyrum dibotrys* (0.57), *Anredera cordifolia* (0.56), *Leucocasia gigantea* (0.52), *Broussonetia papyrifera* (0.52) and *Cinnamomum camphora* (0.52).

**Fig. 7:**
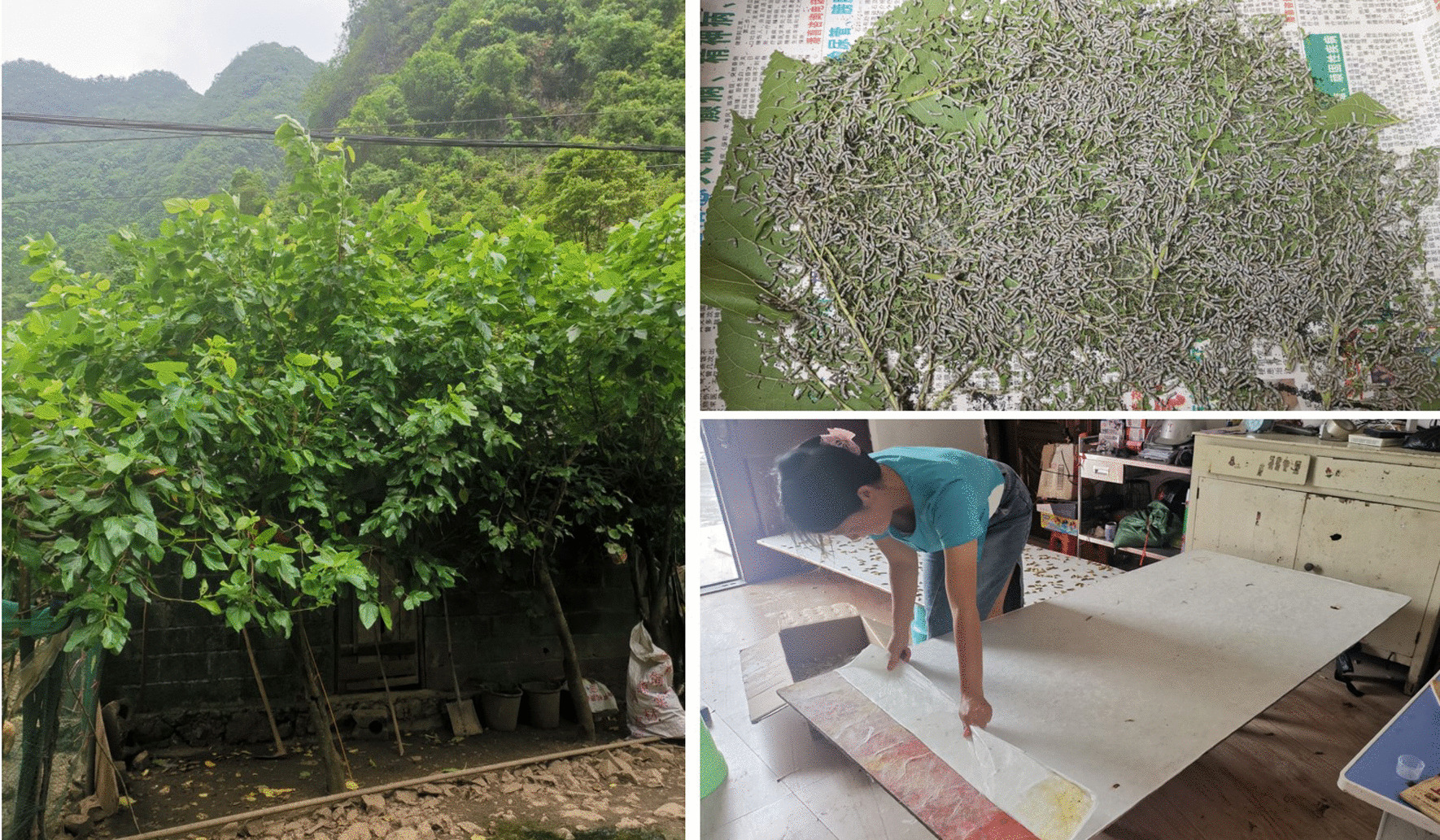
*Morus alb*a and the sericulture of Baiku Yao

*Zea mays* is planted by almost every household; therefore, this species has the highest RFC value. Baiku Yao lives in mountainous areas with minimum landholding, and they grow *Zea mays* as both a staple food and forage because of its high adaptability to such areas and high-yield production. As an important feed plant, *Morus alba* is mainly used to raise silkworms and can also be fed to pigs and cattle. Baiku Yao retains the traditional “farming and weaving culture” in the local area. Their traditional ethnic costumes are all woven from the silk. Therefore, as the only food source for sericulture, *Morus alba* also has a significant local position and value (Fig. 7). *Eriobotrya japonica* has the third-highest RFC value and is utilized for fruit and medicinal purposes (treating cough).

*Ipomoea batatas*, *Solanum melongena*, *Glycine max*, *Phaseolus vulgaris*, *Anredera cordifolia*, *Brassica rapa* var. *oleifera*, *Amaranthus tricolor*, *Cucurbita moschata*, and *Leucocasia gigantea* also have relatively high RFC values. These species are the most common vegetables in the Baiku Yao area for daily needs.

During the interviews, local people said they liked to boil vegetables and meat instead of stir-frying; thus, they could avoid using cooking oil and preserve food nutrition as much as possible. In the past, because of transportation limitations and the economy, Baiku Yao seldom used seasonings such as soy sauce, monosodium glutamate, and salts. They still prefer cultivating various spice species such as *Capsicum annuum*, *Agastache rugosa*, *Cannabis sativa*, *Zingiber officinale,* and *Allium sativum* for more diverse flavors.

As a traditional farming ethnic group, Baiku Yao has a limited income source, and the meat supply mainly comes from self-raised poultry and livestock. Therefore, they grow various fodder species, such as *Zea mays*, *Morus alba*, *Fagopyrum dibotrys*, and *Broussonetia papyrifera,* in their homegardens.

Generally, the homegardens of different villages differ in species preference and utilization patterns. For example, the homegardens of Yaoshan Village and Duonu Community have a higher frequency of ornamental plants such as *Ficus microcarpa*, *Bougainvillea glabra*, and *Zoysia japonica*, but very few people grow these species in Huaili and Yao Villages. However, Huaili and Yaozhai villagers prefer to grow forage species in their homegardens, such as *Broussonetia papyrifera* and *Fagopyrum dibotrys*. These differences in species preferences among the studied villages indicate that they maintained some of the most commonly used species but also introduced various new species to their homegardens after relocation.

**Fig. 8:**
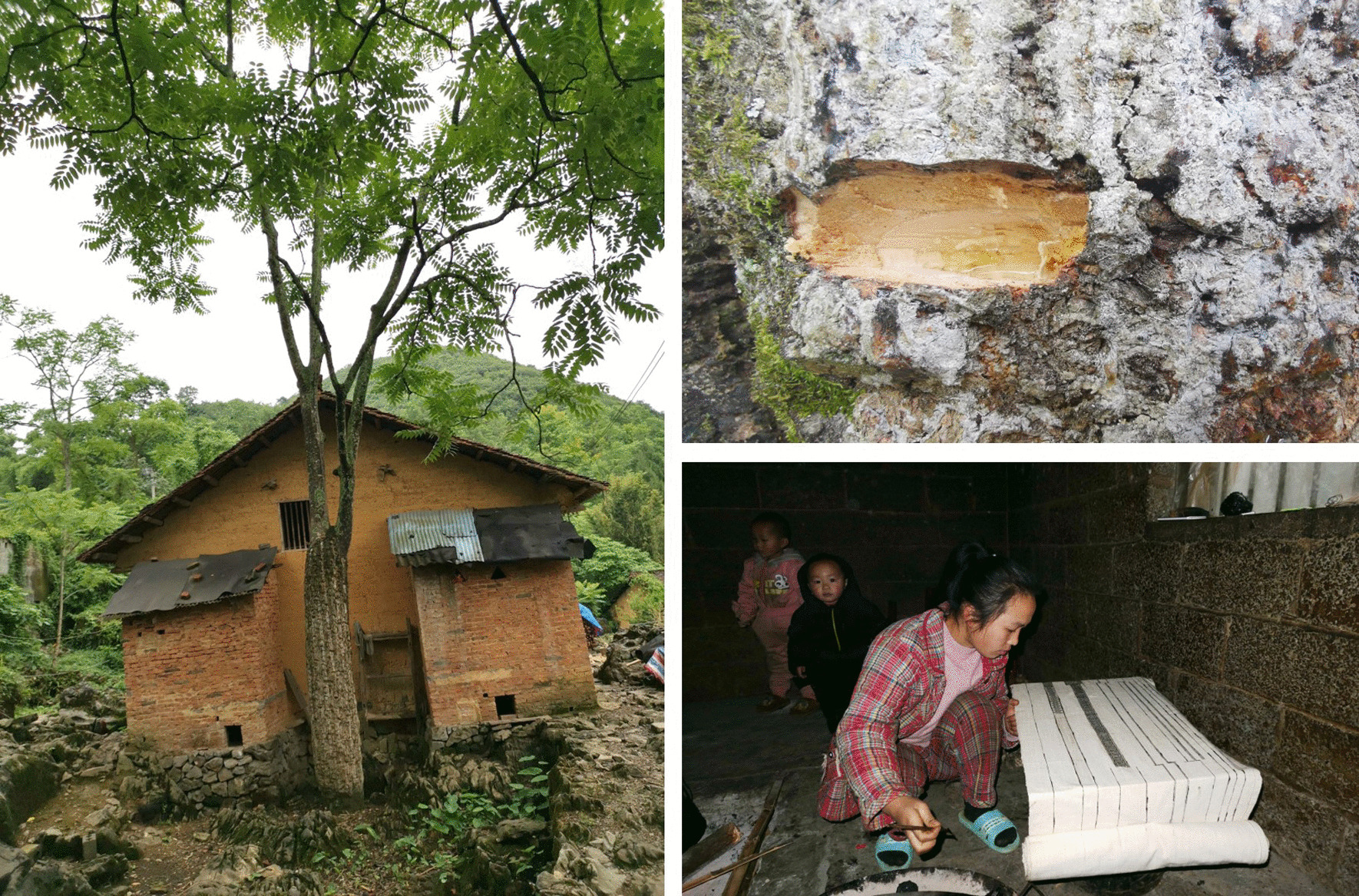
*Ailanthus vilmoriniana* Dode and its resin application

*Ailanthus vilmoriniana*, the totem tree of Baiku Yao, has an important cultural status in the local area. The resin of *Ailanthus vilmoriniana* is an anti-staining agent locally used in the dyeing and patterning of the traditional clothing of Baiku Yao (Fig. 8). Among the villages surveyed, *Ailanthus vilmoriniana* is only distributed in the homegardens of Huaili Village, so its RFC value is very low, yet all the local people of the surveyed villages are very familiar with relevant knowledge of *Ailanthus vilmoriniana*. In fact, market circulation and neighborhood exchanges make up for insufficient *Ailanthus vilmoriniana* resin resources in some villages. In addition, Baiku Yao has a very strong sense of ethnic and cultural identity and often wears traditional costumes, so to a certain extent, it also ensures the preservation of traditional knowledge related to *Ailanthus vilmoriniana*.

## Discussion

### Protection strategy of local homegarden culture

The case of Yaoshan Village can be used as a reference for rural revitalization or tourism development in other regions. While building and developing the local industry, Yaoshan Village pays attention to protecting the traditional livelihood and related culture of the local people, which not only helps the continuation of traditional knowledge but also indirectly protects some special local cultural species. Perhaps, the future development of the Duonu Community can take Yaoshan Village as a template. However, some previous studies have shown that the local industrial structure changed with the gradual development of the regional economy, and these changes altered residents' livelihood and deteriorated the local biodiversity and traditional culture [20–22]. Therefore, attention needs to be paid to maintaining the plant diversity of homegardens by maintaining their ecological and sociocultural functions. Identifying and protecting key species could be one of the most feasible options to protect the traditional knowledge related to homegardens [23]. For example, *Ailanthus vilmoriniana* and some plants with high RFC are likely to be key local cultural species, so their protection should be prioritized [23].

Maintaining the species diversity of homegarden plants can not only help to protect local traditional knowledge but also assist in maintaining the functional diversity of Baiku Yao homegardens to be fully self-sufficient. In the current era, disease epidemics, extreme disasters, and international disputes are more frequent. Responding to the food crisis and sub-disasters has become a prime research interest worldwide. Homegardens have long been reported to be an effective buffer zone for people to resist disasters, and plant diversity and functional diversity are the keys to ensuring social–ecological resilience [24, 25]. Therefore, protecting the plant diversity and functional diversity of Baiku Yao homegardens should be prioritized.

### The traditional knowledge change when migrating

In this case, we can find the difference in the traditional knowledge practice in homegardens between the ancient villages and the relocated ones, brought mainly by the relocation. Traditional knowledge is not immutable but changes dynamically because of environmental change and social development [26]. Historically, there are often cases of population migration around the world, many of them for better living resources and climate or to avoid wars and natural disasters, and the relevant traditional knowledge changes dynamically [27]. For example, in the process of moving south from northern China, the culture of one of the largest migrant groups in history, the Hakka people, blended with the She, Yao, and other ethnic groups [28]. The separation of Hakkas from their original natural resources and cultural atmosphere in the process of migration brought new changes to the traditional experience handed down from generation to generation [28]. The change in traditional knowledge is not necessarily negative, and it may manifest in the local people's adaptation to the new environment and the growth rate of local development.

In many parts of the world, to improve the lives of local people or protect the ecology, local governments are currently building infrastructures and conducting ecological migrations or relocations to make local life more stable and safer [29–31]. However, interfering with the original local livelihood, like banning all wild collection or dramatic environmental changes caused by the long-distance migration, could threaten many traditional practices and lead to cultural damage [31]. However, biodiversity and cultural diversity are inextricably linked [32]. For example, when the species that are available and recognized by local people are significantly reduced in the new environment, the corresponding experience, language, and stories gradually disappear over time. Thus, changes in the original livelihood may also bring negative impact, which needs to be carefully considered by the local government in decision-making.

## Conclusion

We selected two ancient (Huaili and Yaozhai) and two relocated villages (Yaoshan and Duonu) in the Baiku Yao area of China for ethnobotanical investigation. The results show that the traditional knowledge of homegarden plants in Huaili and Yaozhai villages is well preserved, showing good plant diversity and versatility. Due to frequent exchanges between the village and the outside world, Yaoshan Village preserves a good traditional culture and adds many new plants to its homegardens, especially ornamental plants. As a newly relocated village with a short history, Duonu Community has lost some traditional knowledge related to homegarden plants. In addition, the study found that plants such as *Zea mays*, *Morus alba*, and *Capsicum annuum* are the most important in the Baiku Yao homegardens and are closely related to local life and livelihood. In the future development of Baiku Yao communities, protecting homegarden plants and functional diversity is crucial.

## Data Availability

All data generated or analyzed during this study are included in this published article.
